# Metabolic and anatomical predictors of outcome in pulmonary contusion: lessons from a 355-patient cohort

**DOI:** 10.1007/s13304-026-02522-z

**Published:** 2026-02-06

**Authors:** Demet Yaldız, Güntuğ Batıhan, Arkın Acar, Rabia Günseli Emül

**Affiliations:** 1 Department of Thoracic Surgery, Manisa Celal Bayar University, Manisa, Turkey; 2 Department of Thoracic Surgery, Canakkale Onsekiz Mart University, Çanakkale, Turkey

**Keywords:** Pulmonary contusion, Thoracic trauma, Injury severity score, Mortality predictors

## Abstract

Pulmonary contusion is a common consequence of thoracic trauma and is associated with respiratory compromise and increased intensive care demand. However, prognostic determinants in these patients remain incompletely defined. This study aimed to identify clinical and laboratory predictors of in-hospital mortality among trauma patients with pulmonary contusion. We retrospectively analyzed 355 adult trauma patients with pulmonary contusion confirmed by thoracic CT. Demographic, clinical, and laboratory variables were compared between survivors and non-survivors. The Injury Severity Score (ISS) was calculated, and an optimal cut-off was identified using ROC analysis. Independent predictors of mortality were determined using a parsimonious multivariate logistic regression model restricted to clinically relevant and non-collinear variables. The mean age of the cohort was 41.0 ± 17.1 years, and most patients were male (86.5%). Blunt trauma accounted for 86.5% of injuries. Mechanical ventilation was required in 36.3%, and 39.4% were admitted to the ICU. Overall in-hospital mortality was 5.1% (n = 18). In the multivariate model, age (adjusted OR 1.04; 95% CI 1.001–1.079; *p* = 0.043), ISS > 22 (adjusted OR 5.75; 95% CI 1.43–23.13; *p* = 0.014), and serum bicarbonate < 18.8 mmol/L (adjusted OR for survival 0.05; 95% CI 0.01–0.17; *p* < 0.001) remained independent predictors of mortality. Age, admission bicarbonate level, and overall injury severity are independent predictors of in-hospital mortality in thoracic trauma patients with pulmonary contusion. Combining a readily available metabolic marker with global injury severity scoring may enhance early risk stratification and guide clinical decision-making.

**Supplementary Information:**

The online version contains supplementary material available at https://doi.org/10.1007/s13304-026-02522-z.

## Introduction

Pulmonary contusion represents one of the most frequent injuries following blunt thoracic trauma and continues to be a notable cause of morbidity and mortality. Pathologically, it consists of intra-alveolar bleeding and interstitial edema without frank parenchymal laceration, typically associated with rib fractures or high-energy chest impacts. Radiologic manifestations may be inconspicuous in the early phase, yet clinical deterioration can occur rapidly, leading to impaired gas exchange, ventilatory failure, and extended intensive care requirements [1–3].

Despite advances in modern imaging and intensive care strategies, the prognostic determinants among patients with pulmonary contusion remain incompletely defined. While some investigations describe isolated contusions as relatively benign, other reports have linked them to a markedly increased risk of respiratory complications, particularly in the presence of severe or multiple thoracic injuries [4–7].

The present study was designed to evaluate demographic, clinical, and biochemical factors associated with in-hospital mortality in trauma patients diagnosed with pulmonary contusion. We aimed to identify prognostic variables that may improve early risk stratification and guide clinical decision-making in this high-risk patient population.

## Materials and methods

### Study design and setting

This retrospective cohort study was carried out at a tertiary referral trauma center. Adult patients admitted with thoracic trauma were evaluated. Clinical information and imaging data were retrieved from the hospital’s electronic health records and radiology archive systems.

### Patient selection

This retrospective study included adult patients diagnosed with pulmonary contusion following thoracic trauma. The diagnosis was established on chest computed tomography (CT), defined as alveolar opacification without evidence of parenchymal laceration, in conjunction with compatible clinical findings.

#### Inclusion criteria

- Age 18 years or older.
- Admission due to blunt or penetrating chest trauma.
- Availability of thoracic CT imaging at presentation.
- Presence of alveolar opacification consistent with pulmonary contusion.
- Complete clinical and laboratory data.

#### Exclusion criteria

- Isolated head or abdominal trauma without chest involvement.
- Patients with end-stage or functionally severe chronic lung disease—such as oxygen-dependent COPD or advanced fibrotic lung disease—that could obscure or interfere with the radiologic evaluation of pulmonary contusion.
- Missing essential clinical or laboratory outcomes.

All patients were managed according to institutional trauma protocols. Analgesia followed a standardized multimodal regimen; fluid resuscitation adhered to restrictive crystalloid strategies; ventilatory support used lung-protective settings (tidal volume 6–8 mL/kg, PEEP titrated to oxygenation); and chest pathologies (pneumothorax/hemothorax) were treated according to ATLS guidelines [8]. These institutional protocols did not differ between survivors and non-survivors.

### Variables and definitions

For each patient, demographic data (age, sex) and clinical parameters (intensive care unit length of stay, total hospital stays, number of rib fractures, number of pulmonary lobes involved, duration of mechanical ventilation, and need for thoracic surgery) were recorded.

Trauma severity was assessed using the Injury Severity Score (ISS) based on the Abbreviated Injury Scale (AIS, 2005 update) [9]. To determine the optimal cut-off value of the Injury Severity Score (ISS) for predicting in-hospital mortality, receiver operating characteristic (ROC) curve analysis was performed. The threshold was selected using the Youden index.

All laboratory parameters (pH, lactate, bicarbonate) were obtained at initial emergency department presentation within the first hour of admission.

### Statistical analysis

All statistical analyses were performed using IBM SPSS Statistics (version 25.0). The Shapiro–Wilk test was used to assess normality. Continuous variables were expressed as mean ± standard deviation (SD) or median with interquartile range (IQR), depending on distribution. Group comparisons were carried out using the independent-samples t-test or Mann–Whitney U test for continuous variables, and chi-square or Fisher’s exact test for categorical variables.

Variables associated with in-hospital mortality at *p* < 0.05 in univariate analysis, together with clinically relevant covariates identified a priori from the literature, were considered for inclusion in multivariate logistic regression. Given the limited number of events (n = 18), model complexity was restricted to avoid overfitting, following the recommended events-per-variable principle. A parsimonious model was constructed by prioritizing variables with strong biological plausibility, minimal collinearity, and adequate contribution to model fit. Odds ratios (ORs) with 95% confidence intervals (CIs) were reported. A two-sided *p* < 0.05 was considered statistically significant.

## Results

### Patient characteristics

A total of 355 patients fulfilled the study criteria. The mean age was 41.0 ± 17.1 years (range, 16–94), and the majority were male (n = 307, 86.5%). The predominant mechanism of injury was blunt trauma (n = 307, 86.5%), followed by penetrating injuries (n = 37, 10.4%) and crush accidents (n = 11, 3.1%). Rib fractures were present in 216 patients (60.8%); 66 (18.6%) of these were bilateral and 9 (2.5%) were complicated by flail chest. Pneumothorax occurred in 155 cases (43.7%), while hemothorax was observed in 102 (28.7%). Extrathoracic trauma was identified in 297 patients (88.9%). Mechanical ventilation (MV) was required in 129 patients (36.3%), and 140 (39.4%) were admitted to the intensive care unit (ICU). The mean ISS for the cohort was 19.3 ± 7.8 (range, 9–43). Overall, in-hospital mortality was 5.1% (n = 18). Characteristics of the patients were summarized in Table 1.

**Table 1:** Baseline characteristics of patients

Variables	n = 355
Age, years, mean ± SD	41.0 ± 17.1
Sex (male), n (%)	307 (86.5)
Comorbidity (yes), n (%)	86 (24.2)
*Mechanism of Injury, n (%)*	
Blunt	307 (86.5)
Penetrating	37 (10.4)
Crush	11 (3.1)
Rib fracture (yes), n (%)	216 (60.8)
*Number of fractured ribs, mean ± SD*	3.21 ± 0.20
Side of rib fracture, n (%)	
Unilateral	150 (42.3)
Bilateral	66 (18.6)
Flail chest (yes), n (%)	9 (2.5)
Hemothorax (yes), n (%)	102 (28.7)
*Side of lung contusion, n (%)*	
Right	58 (16.3)
Left	58 (16.3)
Bilateral	239 (67.3)
Pneumothorax (yes), n (%)	155 (43.7)
Chest tube insertion (yes), n (%)	119 (33.5)
Extrathoracic trauma (yes), n (%)	297 (88.9)
Need for non-thoracic surgical intervention (yes), n (%)	199 (60.1)
Thoracic surgical intervention (yes), n (%)	43 (13)
ICU (yes), n (%)	140 (39.4)
MV need (yes), n (%)	129 (36.3)
MV duration, days, mean ± SD	5.81 ± 0.66
Hospital stays, days, mean ± SD	19.9 ± 1.45
Contusion healing, days, mean ± SD	9.06 ± 0.40
In-hospital mortality (yes), n (%)	18 (5.1)

*SD* standard deviation, *ICU* intensive care unit, *MV* mechanical ventilation

### Clinical outcomes and pulmonary contusion characteristics

Patients with bilateral lung contusions had significantly longer hospital stays (22.3 ± 29.7 vs. 15.4 ± 17.3 days, *p* = 0.025) and ICU stays (15.9 ± 23.1 vs. 11.2 ± 12.4 days, *p* = 0.045) compared with those with unilateral injuries, although MV duration was not significantly different (6.3 ± 12.1 vs. 4.6 ± 8.6 days, *p* = 0.24).

When stratified by the extent of lung involvement, contusions affecting five lobes were associated with the worst outcomes, including the longest hospital stays (39.1 ± 42.4 days, *p* = 0.004), prolonged ICU stays (26.0 ± 21.1 days, *p* = 0.005), and extended MV duration (11.8 ± 11.9 days, *p* = 0.005). Grouped analysis revealed that patients with ≥ 3 lobes involved had significantly longer hospital stays (24.7 ± 29.9 vs. 16.4 ± 22.8 days, *p* = 0.005), ICU stays (19.2 ± 25.8 vs. 10.7 ± 14.0 days, *p* < 0.001), and MV duration (8.9 ± 14.2 vs. 3.6 ± 7.6 days, *p* < 0.001) compared to those with 1–2 lobes affected (Suppl. 1).

In total, 43 trauma patients required thoracic surgical intervention for diverse indications. The most frequent procedures included thoracic exploration and intrathoracic hematoma evacuation (n = 20), followed by rib fixation and repair of associated chest wall injuries (n = 12). Several patients additionally underwent diaphragm repair, lung parenchymal suturing, or VATS-guided hematoma evacuation and hemostasis (n = 11). Multisystem trauma was common, with combined procedures involving vascular repair, mesenteric or gastrointestinal repair, bladder repair, splenectomy, and orthopedic fixation of the clavicle, pelvis, radius–ulna, or femur. A small number of patients required thoracic laminectomy, and one patient underwent pneumonectomy. Overall, the cohort demonstrated a high burden of complex polytrauma necessitating coordinated thoracic, abdominal, vascular, and orthopedic surgical management.

Among the 18 patients who died, the median time to death was 14.5 days in both ICU and total hospital stay (IQR 6–22.8 and 5.3–27.5 days, respectively). The leading causes were refractory respiratory failure (n = 10), hemorrhagic shock (n = 4), and septic complications (n = 4).

### Comparison of survivors and non-survivors

Non-survivors were significantly older than survivors (50.7 ± 16.9 vs. 40.5 ± 16.9 years, *p* = 0.013). Mortality was higher in patients with ≥ 3 rib fractures (8.2% vs. 2.2%, *p* = 0.009), bilateral rib fractures (13.4% vs. 4.0%, *p* = 0.002), and flail chest (33.3% vs. 4.3%, *p* < 0.001).

Pulmonary contusions involving three or more lobes were associated with increased mortality compared to those affecting one or two lobes (8.3% vs. 2.8%, *p* = 0.021). The need for MV was also strongly associated with fatal outcome (14.0% vs. 0%, *p* < 0.001).

**Fig. 1:**
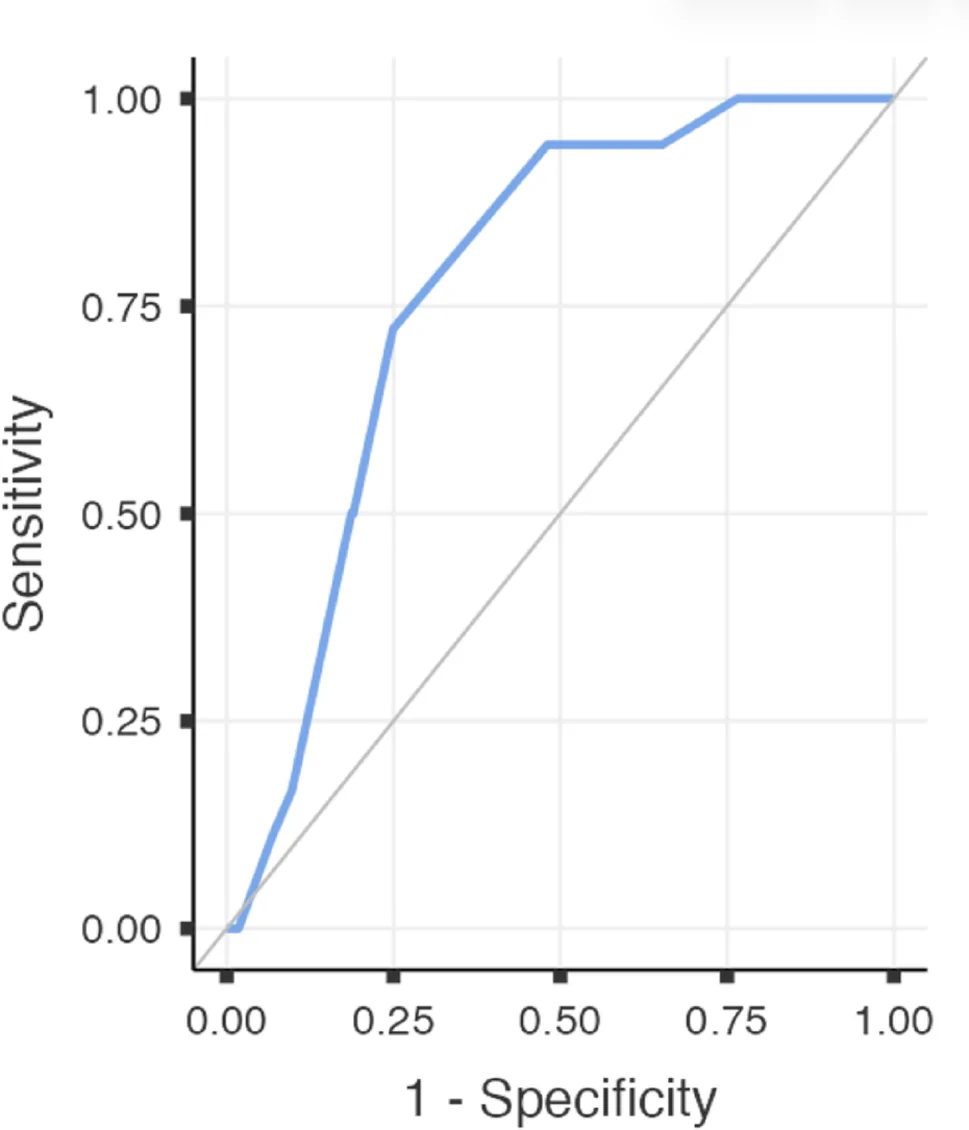
ROC curve of the trauma score for predicting in-hospital mortality. The trauma score demonstrated good discriminative performance with an area under the curve (AUC) of 0.774 (95% CI 0.693–0.855; *p* < 0.001). The optimal cut-off value identified by the Youden index was 22, corresponding to a sensitivity of 72.2% and a specificity of 75.1%

ROC analysis showed that the trauma score performed well in distinguishing survivors from non-survivors, with an AUC of 0.774 (95% CI 0.693–0.855; *p* < 0.001). Based on the Youden index, a score of 22 emerged as the optimal threshold for predicting in-hospital mortality. Using this cut-off, a score ≥ 22 identified mortality with a sensitivity of 72.2% (95% CI 46.5–90.3) and a specificity of 75.1% (95% CI 70.1–79.6) (Fig. 1) (Table 2).

**Table 2:** Comparison of in-hospital mortality across categorical variables

Variables	Mortality, n (%)	*p* value
Number of fractured ribs		0.009
0–2	4/185 (2.2)	
≥ 3	14/170 (8.2)	
Side of rib fracture		0.002
None	3/138 (2.2)	
Unilateral	6/150 (4.0)	
Bilateral	9/67 (13.4)	
Flail chest		< 0.001
No	15/346 (4.3)	
Yes	3/9 (33.3)	
Pleural complication		0.22
No	6/168 (3.6)	
Yes	12/187 (6.4)	
Extent of pulmonary contusion (lobes involved)		0.021
1–2	6/211 (2.8)	
≥ 3	12/144 (8.3)	
Side of lung contusion		0.95
Unilateral	6/116 (5.2)	
Bilateral	12/239 (5.0)	
MV support		< 0.001
No	0/226 (0.0)	
Yes	18/129 (14.0)	
Extrathoracic trauma		0.124
No	0/37 (0.0)	
Yes	18/297 (6.1)	
ISS		< 0.001
≤ 22	5/258 (1.9)	
> 22	13/97 (13.4)	

*MV* mechanical ventilation, *ISS* injury severity score

Laboratory findings at presentation showed that non-survivors had lower pH (7.29 ± 0.20 vs. 7.41 ± 0.11, *p* < 0.001) and bicarbonate levels (19.7 ± 7.4 vs. 24.0 ± 4.2 mmol/L, *p* < 0.001), as well as higher lactate levels (5.4 ± 4.6 vs. 1.9 ± 2.2 mmol/L, *p* < 0.001) (Table 3).

**Table 3:** Comparison of continuous variables between patients with and without in-hospital mortality

Variables	Survivors (n = 337)	Non-survivors (n = 18)	*p* value
Age, years	40.5 ± 16.9	50.7 ± 16.9	0.013
ISS	19.0 ± 7.8	25.6 ± 4.7	< 0.001
SpO₂, %	95.4 ± 7.3	91.4 ± 19.4	0.088
pH	7.41 ± 0.11	7.29 ± 0.20	< 0.001
pO₂, mmHg	112.5 ± 42.9	127.4 ± 62.4	0.280
pCO₂, mmHg	37.8 ± 7.7	39.9 ± 14.2	0.360
HCO₃, mmol/L	24.0 ± 4.2	19.7 ± 7.4	< 0.001
Lactate, mmol/L	1.9 ± 2.2	5.4 ± 4.6	< 0.001

*SpO*_*2*_ Peripheral oxygen saturation (%), *pO*_*2*_ partial pressure of oxygen (mmHg),* pCO *_*2*_Partial pressure of carbon dioxide (mmHg), *HCO*_*3*_ Bicarbonate (mmol/L), *Lactate* Serum lactate (mmol/L)

### Multivariate logistic regression

Variables showing a *p* value < 0.05 in univariate analysis and those considered clinically relevant for model integrity were entered into the multivariate logistic regression. Increasing age (OR 1.04; 95% CI 1.001–1.079; *p* = 0.043), an Injury Severity Score (ISS) > 22 (OR 5.75; 95% CI 1.43–23.13; *p* = 0.014), and serum bicarbonate < 18.8 mmol/L (OR 0.05; 95% CI 0.01–0.17; *p* < 0.001) emerged as independent predictors of in-hospital mortality (Table 4). Although extensive pulmonary contusion involving three or more lobes was associated with higher mortality on univariate analysis, it did not retain statistical significance after adjustment (OR 3.17; 95% CI 0.76–13.26; *p* = 0.11).

**Table 4:** Multivariate logistic regression analysis for predictors of in-hospital mortality

Variables	OR (95% CI)	*p* value
Age	1.04 (1.001–1.079)	0.043
*Extent of pulmonary contusion (lobes involved)*		
1–2	1.00 (reference)	
≥ 3	3.17 (0.76–13.26)	0.11
*ISS*		
≤ 22	1.00 (reference)	
> 22	5.75 (1.43–23.13)	0.014
*HCO*_*3*_, mmol/L		
≥ 18.8	1.00 (reference)	
< 18.8	0.05 (0.01–0.17)	< 0.001

*ISS* injury severity score, *HCO*_*3*_ Bicarbonate (mmol/L), *Lactate* serum lactate (mmol/L)

## Discussion

In this retrospective analysis of 355 patients with blunt thoracic trauma, increasing age, high Injury Severity Score (ISS > 22), and low serum bicarbonate (HCO_3_ < 18.8 mmol/L) emerged as independent predictors of in-hospital mortality. Among these, low bicarbonate showed the strongest association with adverse outcome, highlighting the role of early metabolic acidosis as a marker of physiological compromise [10, 11]. The prognostic impact of age further reflects the reduced physiological reserve and limited compensatory capacity in older trauma patients, consistent with prior reports linking advanced age to higher mortality in thoracic trauma [12–15]. The additional predictive strength of ISS emphasizes the need to consider both anatomical injury burden and systemic physiological response when assessing risk [16–18].

When examined specifically in patients with pulmonary contusion, our findings align with previous studies demonstrating that the extent and distribution of parenchymal injury are strongly correlated with clinical burden and morbidity [1–7]. Patients with bilateral or ≥ 3 lobe involvement experienced significantly longer hospital stays, greater ICU demand, and higher need for mechanical ventilation. However, after adjusting for overall trauma severity and metabolic status, contusion extent no longer showed an independent association with mortality. This pattern suggests that radiologic severity contributes to outcome mainly through secondary physiological disturbances—such as hypoxemia, inflammation, and acidosis—rather than through direct structural damage alone.

The relationship between pulmonary contusion and metabolic derangement underscores the close interplay between local injury and systemic response. The contused lung promotes alveolar flooding, ventilation–perfusion mismatch, and inflammatory mediator release, which collectively contribute to metabolic acidosis and hemodynamic instability [3, 5, 6, 19–21]. In this context, serum bicarbonate serves as a readily available marker of global physiological stress, integrating both pulmonary and extrapulmonary effects. By combining bicarbonate with ISS, clinicians may better identify patients at risk of early deterioration, even before radiologic progression or overt respiratory failure occurs.

From a clinical perspective, these results offer a simple, evidence-based framework for early risk stratification in thoracic trauma with pulmonary contusion. Patients presenting with advanced age, low bicarbonate, or ISS > 22 should be closely monitored, prioritized for early ICU admission, and managed with proactive ventilatory and hemodynamic support. Conversely, patients with limited contusion and preserved metabolic parameters may be safely managed under careful observation.

In summary, this study refines the prognostic understanding of pulmonary contusion by integrating age, anatomical injury, and metabolic derangement into a unified model. Combining an accessible physiological marker (bicarbonate) with a validated anatomical score (ISS) and patient age provides a practical and clinically meaningful approach to early mortality prediction and management in blunt thoracic trauma.

## Limitations

This study has several limitations. Its retrospective, single-center design introduces potential selection bias and limits adjustment for confounders such as injury mechanism, comorbidities, and treatment variations. The small number of mortality events (n = 18) restricted the complexity and statistical power of multivariate analysis, necessitating a focused model to avoid overfitting. Detailed management data—including ventilator settings, resuscitation strategies, and postoperative complications—were unavailable. Finally, these findings require validation in larger, prospective multicenter cohorts to confirm their external applicability.

## Conclusion

In summary, our findings demonstrate that age, admission serum bicarbonate, and Injury Severity Score (ISS) are independent predictors of in-hospital mortality in patients with pulmonary contusion following thoracic trauma. Older patients and those with higher ISS values exhibited an increased risk of death, while low serum bicarbonate levels (< 18.8 mmol/L) served as a strong physiological indicator of poor outcome. Although the extent and distribution of pulmonary contusion were closely related to prolonged hospitalization, mechanical ventilation, and intensive care demand, their prognostic effect on mortality appeared to be mediated through systemic physiological deterioration and overall trauma burden rather than the radiologic extent itself. Incorporating both easily obtainable parameters—patient age, a simple metabolic marker, and a global anatomical severity score—may offer a practical and reliable framework for early risk stratification and informed clinical decision-making in thoracic trauma.

## Supplementary Information

Below is the link to the electronic supplementary material.

## Data Availability

The data that support the findings of this study are derived from institutional medical records and contain sensitive patient information. Due to ethical and legal restrictions, the datasets are not publicly available. De-identified data may be made available from the corresponding author upon reasonable request and subject to approval by the local ethics committee.
