# Supplementary material for: Metabolic and anatomical predictors of outcome in pulmonary contusion: lessons from a 355-patient cohort
Source: Updates Surg. 2026 Feb 6;78(5):2263–9. doi: 10.1007/s13304-026-02522-z (PMC13538228; doi:10.1007/s13304-026-02522-z)
Supplement: Supplementary file 1 [file 13304_2026_2522_MOESM1_ESM.docx]

**Supplement**

**Suppl 1.** Association between extent of pulmonary contusion and clinical outcomes.

| Variables | n (%) | Hospital stay (days), mean±SD | p-value | ICU stay (days), mean±SD | p-value | MV need, (days) mean±SD | p-value |
| --- | --- | --- | --- | --- | --- | --- | --- |
| Side of pulmonary contusion |  |  |  |  |  |  |  |
| Unilateral | 116 (32.6) | 15.4±17.3 |  | 11.2±12.4 |  | 4.6±8.6 |  |
| Bilateral | 239 (67.3) | 22.3±29.7 | 0.025 | 15.9±23.1 | 0.045 | 6.3±12.1 | 0.24 |
| Extent of pulmonary contusion (lobes involved) |  |  |  |  |  |  |  |
| 1 | 56 (15.8) | 12.9±11.1 |  | 9.8±11.5 |  | 3.6±9.1 |  |
| 2 | 155 (43.7) | 17.8±25.9 |  | 11.1±14.9 |  | 3.5±7.1 |  |
| 3 | 97 (27.3) | 21.6±21.9 |  | 17.0±20.9 |  | 7.2±13.4 |  |
| 4 | 31 (8.7) | 26.9±40.5 |  | 22.4±38.6 |  | 12.9±16.8 |  |
| 5 | 16 (4.5) | 39.1±42.4 | 0.004 | 26.0±21.1 | 0.005 | 11.8±11.9 | 0.005 |
| Extent of pulmonary contusion (grouped) |  |  |  |  |  |  |  |
| 1-2 lobes | 211 | 16.4±22.8 |  | 10.7±14.0 |  | 3.6±7.6 |  |
| ≥3 lobes | 144 | 24.7±29.9 | 0.005 | 19.2±25.8 | <0.001 | 8.9±14.2 | <0.001 |

SD: Standard Deviation; ICU: Intensive Care Unit; MV: Mechanical Ventilation.
